# Self-efficacy, motivation and anxiety in novice podiatry students

**DOI:** 10.1186/1757-1146-6-S1-O5

**Published:** 2013-05-31

**Authors:** Ryan Causby, Susan Hillier, Lloyd Reed, Michelle McDonnell

**Affiliations:** 1 School of Health Sciences, University of South Australia, Adelaide, South Australia, 5074, Australia; 2 School of Clinical Sciences, Queensland University of Technology, Brisbane, Queensland, 4064, Australia

## Background

Performance in learning has been linked to a number of factors, including trait-like differences and state-like individual differences such as self-efficacy and anxiety. The aims of this study were to identify the initial level of self-efficacy, motivation and anxiety experienced by students regarding learning scalpel technique and then to identify how this may change following a period of learning.

## Methods

Participants were recruited from the 2^nd^ year cohorts at the University of SA (UniSA) and Queensland University of Technology (QUT). The Intrinsic Motivation Inventory (IMI) was used to evaluate ‘perceived competence’, ‘effort’ and ‘pressure-tension’ associated with scalpel use. This was implemented prior to students learning scalpel use and then again after a period of exposure to public clinics. Scores for each of these factors were calculated. Paired t-tests were undertaken on scores pre- and post- scalpel learning.

## Results

27 students were recruited, 21 from UniSA and 6 from QUT. The mean age of the cohort was 21.4 ± 2.98 years old. None of the students had used a scalpel previously. A mean period of 109 ± 54 days was held between implementation (3 clinics at UniSA and QUT).

**Table 1:** Mean category values

	Pre-	Post-	P-value
	
Perceived competence	24±6.0	28.4±6.0	.001
Effort/ Importance	29.9±3.9	28.8±5.6	.198
Pressure/ Tension	24±4.7	20.8±4.3	.002

## Conclusion

The IMI determined that during teaching and subsequent use of scalpels students’ ‘perceived competence’ improved and ‘pressure-tension’ reduced. This tool may be used to evaluate the impact of differing teaching methods.

